# Prevalence and antimicrobial susceptibility profile of listeria species from ready-to-eat foods of animal origin in Gondar Town, Ethiopia

**DOI:** 10.1186/s12866-015-0434-4

**Published:** 2015-05-12

**Authors:** Legesse Garedew, Ayele Taddese, Tigist Biru, Seleshe Nigatu, Elias Kebede, Mebrat Ejo, Abraham Fikru, Tamiru Birhanu

**Affiliations:** St. Paul’s Hospital Millennium Medical College, Department of Microbiology, Addis Ababa, Ethiopia; Max Planck Institute for Heart and Lung Research, Department III-Developmental Genetics, Bad Nauheim, Germany; University of Gondar, Faculty of Veterinary Medicine, Gondar, Ethiopia; Hawassa University, School of Veterinary Medicine, Awassa, Ethiopia; University of Montreal, Faculty of Veterinary Medicine, Montreal, Canada

**Keywords:** *Listeria*, prevalence, foods, antimicrobials, susceptibility, Gondar

## Abstract

**Background:**

Listeriosis, mostly caused by *Listeria monocytogenes* species, has become a major concern to public health authorities due to its clinical severity and high mortality rate, particularly in high risk groups. Currently, there is limited information regarding the prevalence and antimicrobial susceptibility profiles of listeria species in ready-to-eat foods of animal origin in Gondar town, Ethiopia. The aim of this study was to determine the prevalence and antimicrobial susceptibility pattern of Listeria species isolated from ready-to-eat food of animal origin from public dinning places in Gondar town, Ethiopia. A cross sectional study on ready-toeat foods of animal origin sampled from major supermarkets, butcher shops, pastry shops, restaurants and hotels was carried out. Culture, biochemical and sugar tests were conducted for listeria species identification and disc diffusion test was performed to study the antimicrobial susceptibility profiles of the isolates.

**Results:**

Out of 384 food samples examined, 96 (25%) were positive for Listeria species. *Listeria monocytogenes* was detected in 24 (6.25%) of the samples. *Listeria monocytogenes* was isolated from cake, raw meat, ice cream, minced beef, fish, unpasteurized milk and pizza in that order from higher to lower rate. Assessment of antimicrobial susceptibility profile of *L. monocytogenes* revealed the presence of four multi-drug resistant isolates. The higher resistance rate was recorded for penicillin, nalidixic acid, tetracycline and chloramphenicol, in decreasing order. All *L. monocytogenes* identified in the current study were sensitive to amoxicillin, cephalothin, cloxacillin, sulfamethoxazole, gentamicin and vancomycin.

**Conclusions:**

The presence of *L. monocytogenes* including drug resistant and multidrug resistant isolates in some ready-to-eat food items is an indicator of the presence of public health hazards to the consumer, particularly to the high-risk groups. Hence awareness creation on food safety and implementation of regulations about the use of drugs in humans and animals is strongly recommended.

**Electronic supplementary material:**

The online version of this article (doi:10.1186/s12866-015-0434-4) contains supplementary material, which is available to authorized users.

## Background

Listeriosis is one of the important emerging zoonotic diseases affecting human health following consumption of contaminated foods of animal origin [1]. Twenty to thirty percent of clinical infections of listeriosis result in death. Among the different species of the genus Listeria, *L. monocytogenes* is the causative agent of listeriosis [2].

Listeria species are ubiquitous in the environment and possess unique physiological characteristics that allow growth at refrigeration temperature [3]. High-risk food items associated with listeriosis are ready-to-eat foods including refrigerated but do not undergo any substantial heat treatment before consumption. Major changes in food production, processing and distribution, increased use of refrigeration as a primary preservation method, changes in the eating habits particularly towards ready-to-eat foods, and an increase in the number of people considered to be at high risk for the disease are suggested as possible reasons for the emergence of human food-borne listeriosis [4].

Previously susceptible *L. monocytogenes* become resistant to antimicrobial drugs currently in use for both human and veterinary medicine [5]. Currently, there is inadequate information regarding the prevalence and antimicrobial susceptibility patterns of Listeria spp. in foods in Ethiopia especially in Gondar. Therefore, the present study was undertaken to determine the prevalence and antimicrobial resistance profiles of Listeria species isolated from ready-to-eat foods of animal origin samples collected from Gondar town, Ethiopia.

## Methods

### Study area and study period

The study was conducted from November 2012 to June 2013 at Gondar town public dinning places. Gondar is located northwest of Ethiopia at latitude and longitude of 12°36’N and 37°28’E. Its altitude is 2200 meters above sea level. The maximum and minimum temperatures of the area are 30.7°C and 12.3°C, respectively. The total human population of the town is estimated to be 206,987. Gondar town, as one of most visited historical place in Ethiopia, hosts large number of visitors including foreign tourists [6]. According to the information obtained from Gondar town trade and industry office in 2013, there are about 273 licensed public dinning establishments including 80 hotels, 18 restaurants, 65 bar and restaurants, 20 pastry and 90 butchery shops.

### Study design and sampling

A cross-sectional study was conducted to determine the prevalence and antimicrobial susceptibility profile of Listeria isolates from foods of animal-origin samples purchased from a randomly selected public dinning houses (cafeterias, hotels, restaurants, pastry and retail shops) in Gondar town. Study samples were collected using simple random sampling based on proportional allocation from a complete list of public dinning places of the town.

Since there was no previous study in the area, sample size was estimated by taking 50% expected prevalence with 95% confidence interval and 5% desired accuracy level [7]. Accordingly a total of 384 food samples consisting of 50 raw and 50 pasteurized milk, 40 cheese, 65 cream cakes, 20 ice cream, 85 minced raw meat, 24 pizza and 50 fish foods were collected aseptically using sterile plastic bags and transported immediately using icebox to microbiology laboratory. Out of each sampling unit that consisted of 100 g, 25 g analytical units were subsequently removed for microbiological analysis.

### Identification of listeria species

Half Fraser broth (AES LAB., Combourg, France) was used as a primary selective enrichment medium. Ferric ammonium citrate (Sigma, Steinheim, Germany) was added as a supplement. Then, 25gm food sample was mixed with 225 ml half Fraser broth in a stomacher bag and homogenised using a laboratory blender, stomacher-400™ (Seward Medical, London, UK) at a higher speed for two minutes and incubated at 30°C for 24 h. Similarly, 25 ml milk was sampled and pH adjusted to neutral and thoroughly mixed with 1:10 ratio to half Fraser broth and incubated at 30°C for 24 h. On the second day, about 0.1 ml of culture was transferred to a tube containing 10 ml of Fraser broth (containing acriflavin hydrochloride 0.025 g/l of distilled water and nalidixic acid 0.02 g/l of distilled water)(AES Lab., Combourg, France) and ferric ammonium citrate. The inoculated medium was incubated at 37° for 48 h. Then a loopful of inoculum was taken from isolated colonies and streaked onto pre-dried sterile plates of PALCAM (Polymixin Acriflavin Lithium Chloride Ceftazidime Aesculin Mannitol). Plates were examined for grey-green colonies with black background, typical for Listeria spp. Selected representative colonies were further identified to species level based on haemolytic patterns and biochemical analysis, following previously described protocol by Rorvik and his associates [8].

For confirmation, from each PALCAM agar plates (BD Diagnostic Systems, Heidelberg/Germany), five colonies presumed to be Listeria species were taken and streaked onto the surface of pre-dried plates of Tryptone Soya Yeast Extract Agar (TSYEA) (Detroit, USA) and incubated at 37°C for 24 h or until growth is satisfactory. Typical colonies (1 mm to 2 mm in diameter, convex, colourless and opaque), were used for further biochemical tests. Gram staining, motility, haemolysis, catalase, and CAMP tests were also performed. Rhamnose (AES Lab., Combourg, France), Xylose (AES Lab., Combourg, France) and Mannitol (Merck, Darmstadt, Germany) fermentation was evaluated and positive reactions (acid formation) were indicated by a yellow colour within 24 to 48 h [9,10].

### Antimicrobial susceptibility testing

Antimicrobial susceptibility test was performed for *L. monocytogenes* isolates using Kirby Bauer disc diffusion technique [11]. About 2–3 pure colonies were taken from TSYEA, suspended in Muller Hinton broth and incubated at 37°C for 1–2 h. Bacterial suspension was then adjusted to 0.5 McFarland turbidity standards and transferred to Mueller-Hinton agar plate using a sterile cotton swab. Plates were seeded uniformly by rubbing the swab against the entire agar surface. After the inoculums were dried, antibiotic impregnated disks were applied to the surface of the inoculated plates using disc dispenser. The plates were then incubated aerobically at 37°C for 24 h. Finally, zone of inhibition was measured including the disk diameter. The susceptible, intermediate and resistant categories were assigned on the basis of the critical points recommended by Clinical and Laboratory Standards Institute (CLSI) [12] and according to the manufacturer’s leaflet attached to the disks. The antimicrobials (Oxoid Ltd, Basingstoke, Hampshlre, England) tested were amoxicillin (AML 20 μg), sulfamethoxazole-trimethoprime (SXT 25 μg), cephalothin (KF 30 μg), chloramphenicol (CAF 30 μg), cloxacillin (OX 30 μg), penicillin (P 10 μg), tetracycline (TE 30 μg), vancomycin (VA 30 μg), and gentamicin (CN 10 μg). Selection of antimicrobials was based on availability and frequent use of these antimicrobials in the study area both in veterinary and human medicine. *L. monocytogenes* ATCC 19111 were used as quality control strain.

### Data management and analysis

Data was entered and analyzed using the statistical software SPSS version 20.0. Descriptive statistics was used to describe the frequency of Listeria species from different food samples, antimicrobial susceptibility pattern and hygienic conditions.

### Ethical clearance

Ethical clearance was obtained from the Ethical Review Board of Institute of Public Health, University of Gondar. Written consent was received from public dinning house owners participated in this study. Confidentiality of the information was maintained throughout the study.

## Results

Out of 384 food samples examined in this study, 96 (25.0%) food samples were contaminated with Listeria species. Listeria species were isolated from raw minced beef, fish meat, pizza, raw milk, cottage cheese, cream cake and ice cream samples as tabulated in Table 1.

**Table 1:** **Prevalence of Listeria species in food items from public dinning places in Gondar**

**Sample type**	**No of Sample** **Examined**	***L. monocytogenes*** **N (%)**	**Other Listeria** **specie N (%)**	**Total** **N (%)**
**Raw meat**	60	4(6.66)	27(45)	31(51.66)
**Minced beef**	25	3(12)	3(12)	6(24)
**Fish meat**	50	3(6)	10(20)	13(26.0)
**Pizza**	24	2(8.3)	5(20.8)	7(29.16)
**Pasteurized milk**	50	0	0	0
**Raw milk**	50	2(4.0)	12(24)	14(25.0)
**Cottage cheese**	40	0	5	5(12.5)
**Ice cream**	20	3(15)	6(30)	9(45)
**Cream Cakes**	65	7(10.7)	4(6.15)	11(16.9)
**Total**	**384**	**24(6.25)**	**72(18.75)**	**96(25 %)**

The highest and the lowest prevalence of Listeria were found in raw meat (31/60, 51.66%) and cottage cheese (5/40, 12.5%). All pasteurized milk (n = 50) samples examined were negative for Listeria species. *L. monocytogenes* were isolated from 24(6.25%) of the total food samples analysed and it was the second predominant Listeria species next to *L. innocua* (12.5%) as indicated in Table 2. The prevalence of *L. innocua* was found to be the highest in raw meat (31.7%) followed by raw milk (14%) and fish meat (10%) samples. In the current study, ice cream was the most contaminated food item (15%) with *L. monocytogenes* followed by minced beef (12%) and cream cakes (10.7%).

**Table 2:** **Distribution of Listeria species by source in Gondar town**

	**Number and percentage of Listeria species isolated from the food items examined**										
**Listeria species** **isolated**	**Raw** **meat (N)**	**Minced** **beef (N)**	**Fish (N)**	**Pizza(N)**	**Pasteurized** **milk (N)**	**Raw** **milk (N)**	**Cottage** **cheese(N)**	**Ice cream(N)**	**Cream** **cake(N)**	**Total (N)**	**Prevalence (%)**
*L.monocytogenes*	4	3	3	2	0	2	0	3	7	24	6.25
*L. ivanovii*	2	0	0	0	0	0	0	0	0	2	0.52
*L. innocua*	19	2	5	4	0	7	3	4	4	48	12.5
*L. seeligeri*	2	0	2	0	0	1	0	0	0	5	1.3
*L. welshimeri*	4	1	1	1	0	2	0	0	0	9	2.3
*L. grayi*	0	0	0	0	0	2	0	0	0	2	0.5
*L. murrayi*	0	0	2	0	0	0	2	2	0	6	1.6
**Total positive**	31	6	13	7	0	14	5	9	11	96	25
**Sample examined**	60	25	50	24	50	50	40	20	65	384	
**Prevalence (%)**	**51.6**	**24**	**26**	**29.1**	**0.0**	**25**	**12.5**	**45**	**16.9**	**25**	

### Antimicrobial susceptibility profile of *L. monocytogenes*

Of the total 24 *L. monocytogenes* species subjected for antimicrobial susceptibility test, 16 (66.7%) exhibited resistance for penicillin. Twelve (50%), 9(37.5%) and 4(16.6%) of *L. monocytogenes* were resistant to nalidixic acid, tetracycline and chloramphenicol, respectively. Of those *L. monocytogenes* species exhibited resistance for one or more antimicrobials, 4(16.7%) were multidrug resistance. Multidrug resistant isolates were identified from raw meat (n = 2), minced beef (n = 1) and fish (n = 1). All of *L. monocytogenes* identified in this research were sensitive to amoxicillin, cephalothin, cloxacillin, sulfamethoxazole-trimethoprime, gentamicin and vancomycin as indicated in Table 3.

**Table 3:** **Antimicrobial resistance profiles of** ***L. monocytogenes*** **from food samples**

	**Number of isolates (%)**		
**Antimicrobial agent**	**Resistant**	**Intermediate**	**Susceptible**
**Amoxicillin**	0	0	24(100%)
**Cephalothin**	0	0	24(100%)
**Chloramphenicol**	4(16.6%)	6(25%)	14(58.3%)
**Cloxacillin**	0	0	24(100%)
**Sulfamethoxazole**	0	0	24(100%)
**Gentamicin**	0	0	24(100%)
**Nalidixic acid**	12(50%)	4(16.6%)	8(33.3%)
**Penicillin**	16(66.7%)	3(12.5%)	5(20.8%)
**Tetracycline**	9(37.5%)	2(8.3%)	13(54.2%)
**Vancomycin**	0	0	24(100%)

## Discussion

The overall prevalence of Listeria species in all food samples examined in this study was 25% that indicated significant public health hazard associated with consumption of contaminated foods of animal origin. The isolation rate of *L. monocytogenes* was comparable with other reports from Ethiopia [13,14]. This finding was also inline with the study done in Chile [15]. Relatively higher prevalence levels of L*. monocytogenes* were reported in foods from Norway [16] and Turkey [17]. Differences in the prevalence of *L. monocytogenes* in foods in different countries might be attributed to differences in food items composition or hygienic status of food processing plants. Furthermore the sensitivity of bacteriological detection methods could partially explain these differences.

During this study, meat and meat product foods showed relatively higher level of contamination with Listeria species. Current isolation of *L. innocua, L. welshimeri* and *L. murrayi* was in agreement with findings of other study carried out in Ethiopia [14]. The prevalence of *L. monocytogenes* from minced beef in the current investigation was comparable with previous report from Ethiopia [13] but higher compared with the finding of a study done in Korea [18].

As per the authors’ best knowledge, *L. monocytogenes, L. innocua* and *L. welshimeri* were isolated from pizza samples for the first time in Ethiopia in the current research. Prevalence of *L. monocytogenes* and other Listeria species from fish samples is in line with the research finding carried out in Japan [19] but higher than the prevalence reported from Ethiopia [20,21] and India [4,8]. The null detection rate of Listeria species from pasteurised milk comply with the previous study done in Addis Ababa, Ethiopia [13]. This might be due to the fact that pasteurisation kills Listeria and absence of contamination following pasteurisation. About 4% prevalence of *L. monocytogenes* from 50 raw milk samples examined was lower compared with 8.3% and 13% prevalence report from Addis Ababa, Ethiopia, respectively [14,20]. This could strengthen the fact that raw milk must be considered by the dairy processor as a source of contamination. This result might be an indication of poor hygienic and sanitary condition of the milk processing and supply chain since the origin of *L. monocytogenes* contamination in milk is mainly of faeces [22,23].

The increased contamination of ice cream samples with *L. monocytogenes, L. innocua* and *L. murrayi* might be due to the nature of the ice cream that provides a suitable environment with regard to pH, water activity, nutrient availability and storage temperatures [24,25]. The prevalence of *L. monocytogenes* in ice cream samples in this study was comparatively higher than a report from Addis Ababa, Ethiopia [13]. This might be an indication of poor hygienic handling and preparation practices of ice cream in the current study settings. It was noted that there were no routine food safety control systems in-place in the study area. In addition, power supply interruption was common in the town which can directly affect the shelf life and hygienic quality of food items sold in the pastry shops and supermarkets [14]. Isolation of *L. monocytogenes* from ice cream and frozen foods indicates its survival at freezing temperature [24] which implies its increased public health significance [21]. In contrary to other studies conducted in Ethiopia and elsewhere in the world [14,21,26–29], the current study revealed no *L. monocytogenes* detection from cottage cheese.

Four (16.7%) *L. monocytogenes* isolates identified in this study showed a multi-drug resistant profile according to the recent multidrug resistance definition [30]. Further analysis of antimicrobial susceptibility test results showed that 87.5% of the isolates were resistant at least for one or more antimicrobials tested. In addition, 66.7% of *L. monocytogenes* species identified were resistant to penicillin which might be ascribed to high level utilization of this antibiotic due to its relatively cheaper price and readily available nature to the local community. Penicillin is one of the most frequently prescribed drugs in the current study area for most of infectious diseases both in veterinary and human medicine that could be mentioned as one of the reasons for the development of such higher resistance profile.

Resistance was also encountered for nalidixic acid, tetracycline and chloramphenicol which were similar with studies conducted in different countries [27,29,31]. The presence of antimicrobial resistant *L. monocytogenes* in raw food products has an important public health implication especially in developing countries where there is widespread and uncontrolled use of antibiotics [31]. The problem can be higher in Ethiopia since consumption of raw meat and milk products is very common and due to the presence of large number of high-risk population. Majority of the *L. monocytogenes* were susceptible to amoxicillin, cephalothin, cloxacillin, sulfamethoxazole-trimethoprime, gentamicin and vancomycin which was in agreement with the finding of an earlier study in Addis Ababa, Ethiopia [14].

## Conclusion

The microbiological examination of ready-to-eat foods of animal origin samples for Listeria species in this study revealed the presence *L. monocytogenes* in varying prevalence with the exception of pasteurized milk and cottage cheese. In addition, drug resistant including multidrug-resistant *L. monocytogenes* were found circulating among ready-to-eat foods of animal origin in Gondar town posing high risk of infection for consumers. Application of hazard analysis critical control point principle and treatment based on *in-vitro* antimicrobial susceptibility tests should be in-place. Health education about the risk of consumption of raw foodstuffs should be implemented. Further in-depth typing and drug resistant gene identification studies are recommended.
